# Interhemispheric Functional and Structural Disconnection in Alzheimer’s Disease: A Combined Resting-State fMRI and DTI Study

**DOI:** 10.1371/journal.pone.0126310

**Published:** 2015-05-04

**Authors:** Zhiqun Wang, Jianli Wang, Han Zhang, Robert Mchugh, Xiaoyu Sun, Kuncheng Li, Qing X. Yang

**Affiliations:** 1 Department of Radiology, Xuanwu Hospital of Capital Medical University, Beijing, China; 2 Center for NMR Research, Department of Radiology, Pennsylvania State University College of Medicine, Hershey, Pennsylvania, United States of America; 3 Center for Cognition and Brain Disorders and the Affiliated Hospital, Hangzhou Normal University, Hangzhou, Zhejiang, China; 4 Key Laboratory for Neurodegenerative Diseases (Capital Medical University), Ministry of Education, Beijing, China; 5 Beijing Key Laboratory of Magnetic Resonance Imaging and Brain Informatics, Beijing, China; 6 Department of Neurosurgery (George M. Leader Foundation Alzheimer’s Laboratory), The Pennsylvania State University College of Medicine, Hershey, Pennsylvania, United States of America; & National Laboratory of Pattern Recognition, CHINA

## Abstract

Neuroimaging studies have demonstrated that patients with Alzheimer’s disease presented disconnection syndrome. However, little is known about the alterations of interhemispheric functional interactions and underlying structural connectivity in the AD patients. In this study, we combined resting-state functional MRI and diffusion tensor imaging (DTI) to investigate interhemispheric functional and structural connectivity in 16 AD, 16 mild cognitive impairment (MCI), as well as 16 cognitive normal healthy subjects (CN). The pattern of the resting state interhemispheric functional connectivity was measured with a voxel-mirrored homotopic connectivity (VMHC) method. Decreased VMHC was observed in AD and MCI subjects in anterior brain regions including the prefrontal cortices and subcortical regions with a pattern of AD<MCI<CN. Increased VMHC was observed in MCI subjects in posterior brain regions with patterns of AD/CN < MCI (sensorimotor cortex) and AD < CN/MCI (occipital gyrus). DTI analysis showed the most significant difference among the three cohorts was the fractional anisotropy in the genu of corpus callosum, which was positively associated with the VMHC of prefrontal and subcortical regions. Across all the three cohorts, the diffusion parameters in the genu of corpus callosum and VMHC in the above brain regions had significant correlation with the cognitive performance. These results demonstrate that there are specific patterns of interhemispheric functional connectivity changes in the AD and MCI, which can be significantly correlated with the integrity changes in the midline white matter structures. These results suggest that VMHC can be used as a biomarker for the degeneration of the interhemispheric connectivity in AD.

## Introduction

Alzheimer's disease (AD), the most common form of dementia, presents with memory and cognitive decline. Mild cognitive impairment (MCI) is considered a precursor of AD; people with MCI are diagnosed with AD at a rate of 10–15% per year [1] compared to 2–3% per year for the general population of the same age range. It is unclear, however, how the AD pathological lesions initiated in the medial temporal lobes lead to prominent functional deficits in memory and ultimately to dementia. Deciphering such structural-functional relationships could shed light into the trajectory of the functional pathogenesis of AD and MCI from normal aging. It has been shown that AD patients can perform normally for some tasks that relied on intrahemispheric processing but perform poorly for the tasks that required interhemispheric communication [2], which suggests a plausible hypothesis that AD patients may present a deficit in the interhemispheric integration of information. This hypothesis is supported by morphologic MRI findings that the corpus callosum, the most important fiber tract for interhemispheric connectivity, has consistently exhibited marked atrophy in AD and MCI [3, 4]. Diffusion tensor imaging (DTI) studies of the corpus callosum have demonstrated significant changes in fractional anisotropy (FA), mean diffusivity (MD), radial diffusivity (λ_┴_) as well as axial diffusivity (λ_‖_) in early AD and MCI [5–7]. Yet, the relationship between these structural changes and the functional deficits remains to be determined [8].

To begin to address this issue, one must have a quantitative measure for inter-hemispheric functional connectivity. Recently, resting-state functional connectivity (RSFC) has been applied to investigate AD and MCI [9–15]. Using regions-of-interest-based functional connectivity approach, reduced functional connectivity related to the hippocampus [9, 10] and posterior cingulate cortex (PCC) [11–13] has been observed in AD and MCI patients. By employing the independent component analysis (ICA) method, researchers have reported widespread disruptions of selective brain networks such as the default mode network (DMN) and attention network in AD and MCI [14,15]. Furthermore, functional connectivity is consistently found across homotopic sites in the two hemispheres [16, 17]. Strong interhemispheric RSFC is a common characteristic of the brain’s intrinsic functional networks such as the DMN, memory and sensorimotor networks [9,18,19]. Thus, the interhemispheric RSFC can be potentially used for assessing the integration of information from the two hemispheres. At present, interhemispheric functional connectivity in AD and MCI remains barely explored.

Recently, a novel voxel-wise image analysis method called voxel-mirrored homotopic connectivity (VMHC) was proposed to assess RSFC between the two hemispheres. VMHC quantifies the RSFC between each voxel in one hemisphere and its mirrored counterpart in the other. Using this method, interhemispheric RSFC was found to increase in the sensorimotor regions and decrease in higher-order cognitive regions during normal aging [20]. Clinically, characteristic patterns of significant VMHC disruptions have been found in autism [21], cocaine addiction [22], schizophrenia [23], multiple sclerosis [24], and depression [25]. These findings suggested that specific patterns of interhemispheric disconnection could reflect the functional consequences of pathologic damages in the associated diseases. Thus, a specific pattern of interhemispheric connectivity change is anticipated in MCI and AD when cognitive impairments are significantly progressed. We hypothesize that there are measureable differences in interhemispheric connectivity in AD and MCI patients compared to the age-matched cognitive normal (CN) healthy subjects. Furthermore, since there should be strong links between structural and functional connectivity in the human brain, we speculate that interhemispheric structural connectivity is disrupted by AD pathology, which will result in the loss of functional connectivity. Recent studies indicate myelin can be directly damaged by amyloid-β plaques that are an important early event in the pathogenesis of AD [26]. To test this hypothesis, we examined the interhemispheric connectivity in AD and MCI functionally using VMHC and structurally using DTI. We aimed to find a progressive VMHC disruption in the specific brain areas that cognitive and memory functions are depended on.

## Materials and Methods

### Subjects

Forty-eight right-handed subjects (16 AD, 16 MCI, and 16 age/gender-matched CN) were recruited and participated in the study at the memory clinic of a neurological institute. The study was approved by local Medical Research Ethics Committee. All subjects provided written informed consent prior to participation, consistent with the Declaration of Helsinki. All the AD and MCI patients underwent a complete physical and neurological examination standard laboratory tests, and an extensive battery of neuropsychological assessments.

The AD patients were clinically diagnosed by the AD specialists in the memory clinic and met the criteria for dementia from the Diagnostic and Statistical Manual of Mental Disorders 4th Edition [27], as well the criteria for possible or probable AD from the National Institute of Neurological and Communicative Disorders and Stroke/Alzheimer Disease and Related Disorders Association (NINCDS-ADRDA) [28].

Participants with MCI had memory impairment but did not meet the criteria for dementia. The criteria for the identification and classification of subjects with MCI were as follows: (a) impaired memory performance on a normalized objective verbal memory delayed-recall test; (b) recent history of symptomatic worsening in memory; (c) normal or near-normal performance on cognitive tests, including a Mini-Mental State Examination (MMSE) score >24, as well as normal activities described in a daily living scale; and (d) global rating of ≤ 0.5 on the Clinical Dementia Rating (CDR) score [29], with a score of at least 0.5 on the memory domain [30, 31]. The diagnosis of MCI fulfilled the new criteria of the MCI due to AD recommended by the National Institute on Aging-Alzheimer‘s Association workgroups [32].

The criteria for CN were as follows: (a) no neurological or psychiatric disorders; (b) no neurological deficiencies; (c) no significant abnormal findings in conventional brain MRI; (d) no cognitive complaints; (e) MMSE score of 28 or higher; and (f) CDR score of 0.

### Data acquisition

MRI data acquisition was performed on a 3-Tesla scanner (Siemens Medical Solutions, Erlangen, Germany). Foam padding and headphones were used to limit head motion and reduce scanner noise. The subjects were instructed to hold still, keep their eyes closed, and think of nothing in particular during resting state fMRI (rs-fMRI) data acquisition. These rs-fMRI images were acquired using an echo-planar imaging (EPI) sequence with a repetition time (TR)/echo time (TE)/flip angle (FA) = 2000 ms/40 ms/90°, field of view (FOV) = 256 mm × 256 mm, acquisition matrix = 64 × 64, 28 axial slices, slice thickness = 4 mm, slice distance = 1 mm, bandwidth = 2232 Hz/pixel, and number of repetitions = 239. The 3D T1-weighted anatomical image was acquired with a magnetization-prepared rapid gradient echo (MPRAGE) method in the following parameters: TR/TE/inversion time (TI) /FA = 1900 ms/2.2 ms/900 ms/9°, FOV = 224 mm × 256 mm × 176 mm, acquisition matrix = 224 × 256 × 176. DTI parameters were: 12 non-linear directions, b-value = 1000 s/mm^2^, TR/TE = 6000 ms /85 ms, 30 axial slices, slice thickness = 5 mm, FOV = 256 mm × 256 mm, acquisition matrix = 128×128, image resolution = 256 × 256, number of averages = 4.

### Resting state fMRI data processing and statistical analysis

The rs-fMRI data were processed using statistical parametric mapping (SPM8) and Data Processing Assistant for Resting-State fMRI (DPARSF) toolkits [33]. The first four images of each rs-fMRI data set were discarded to remove the initial transient signal fluctuations. Subsequent images were corrected for slice-timing and re-aligned within the session to remove any minor head movements. The T1-weighted image was co-registered to the mean rs-fMRI image using rigid-body transformation, and then spatially normalized to the Montreal Neurological Institute (MNI) space using nonlinear transformation and the SPM8 T1 template. The rs-fMRI images were spatially normalized in a spatial resolution of 3 × 3 × 3 mm using the same normalization parameters as the T1 image, then smoothed with an 8 × 8 × 8 mm full width at half maximum (FWHM) Gaussian kernel. Linear detrending and temporal bandpass filtering (0.01–0.08 Hz) were applied to reduce the effect of low-frequency drifts and high-frequency physiological noise. Finally, the effects from several nuisance variables including six head motion parameters, global mean signal, cerebrospinal fluid signal and white matter signal were removed by multiple linear regression analysis.

For VMHC analysis, a left-right hemisphere symmetric brain template was generated from the 48 subjects to remove the geometric differences between the two hemispheres. First, a mean T1 image was generated by averaging the 48 spatially normalized T1 images. Next the symmetric brain template was obtained by flipping the left and right hemispheres along the midline of the x-axis and averaged with the original image to create the final template. Then the T1 image from each individual subject that had been normalized to the MNI space was co-registered nonlinearly to this group-specific symmetric brain template. The same transformation was then applied to the rs-fMRI images. Homotopic RSFC between each pair of symmetric voxels in left-right hemispheres was calculated with a Pearson’s correlation and then Fisher-Z transformed for further statistical analysis. The connectivity values in each pair of voxels were the same. For group analysis, the voxels within a range of x = ± 4.5 mm from the midline were excluded from the VMHC maps to reduce the blurring artifacts near the midline. The average VMHC map from each cohort was evaluated with a voxel-based one-sample t-test [Family-wise error (FWE) corrected, *p* < 0.001, extent threshold = 10]. Group comparisons of VMHC were conducted using a one-way analysis of variance (ANOVA) with age and total grey matter volume as the nuisance covariates (*p* < 0.01, corrected using the AFNI AlphaSim program (http://afni.nimh.nih.gov/pub/dist/doc/manual/AlphaSim.pdf) with a cluster size > 97 voxels, FWHM = 8 mm, and cluster radius connection = 5). The total grey matter volume that used in the data analysis was the ratio between the brain grey matter volume and the intracranial volume from each subject. The brain and intracranial volumes were measured from the segmented T1 images. The intracranial volume equals the total volume of grey matter, white matter, and cerebrospinal fluid.

### DTI data processing and statistical analysis

The DTI data were processed following standard procedure with DTI-Studio version 3.0.3 (Johns Hopkins University, Baltimore, MD). DTI parametric maps (FA, MD, λ_‖_ and λ_┴_) were estimated from each subject, co-registered to the T1-weighted image with the mean B_0_ image as the source image by SPM8, normalized to the MNI space with diffeomorphic anatomical registration using exponential lie algebra (DARTEL) in a spatial resolution of 2 × 2 × 2 mm, and smoothed with a 10 × 10 × 10 mm FWHM Gaussian kernel. The comparison of each diffusion parameter map among the three study cohorts was made using a voxel-based one-way ANOVA with intracranial volume correction. The diffusion parameters of the above-threshold clusters that showing the most significant differences across cohorts were recorded from each subject and then used in further post-hoc analysis with IBM SPSS Statistics for Windows Version 21. Correlation analyses were conducted among regional diffusion parameters, regional VMHCs and MMSE scores for all the subjects and for each subject group. Since age is both a significant risk factor for AD and correlates with diffusion parameters in normal aging [34], age was used as a confounding factor in the correlation analysis.

## Results

### Clinical and neuropsychological examination

Clinical and demographic data of the three cohorts were shown in Table 1. The MMSE, CDR, Montreal Cognitive Assessment (MoCA), Clock Drawing Task (CDT) and Activity of Daily Living scale (ADL) scores were significantly different among the three groups. No significant differences were found in gender, age, education, Hamilton Depression Scale (HAMD), or Hachinski Ischemic Score (HIS) among cohorts.

**Table 1:** Demographic information and neuropsychological test scores.

	AD (n = 16)	MCI (n = 16)	CN (n = 16)	*p*-value
Age (years)	71.56 ± 5.93	70.88 ± 7.61	69.25 ± 7.83	0.648
Gender (M/F)	7/9	7/9	7/9	0.923
Education (years)	9.81± 3.62	10.25 ± 3.92	9.50± 3.54	0.848
CDR	0.84 ± 0.51	0.38 ± 0.22	0 ± 0	<0.001
MMSE	18.00 ± 3.43	26.63 ±1.09	28.63 ± 0.72	<0.001
MoCA	15.56 ± 3.05	24.00 ± 1.93	28.56 ± 0.81	<0.001
CDT	6.06 ± 1.39	7.69 ± 0.60	8.63 ± 0.50	<0.001
ADL	28.63 ± 6.27	22.25 ± 1.13	21.25 ± 0.86	<0.001
HAMD	1.19 ± 1.38	1.06 ± 1.12	0.44 ± 0.63	0.125
HIS	2.00 ± 0.82	1.88 ± 0.96	1.56 ± 0.81	0.346

Data are presented as mean ± std. p-value indicates the significance of the difference among the three study cohorts (one-way ANOVA). Abbreviations: CDR, Clinical Dementia Rating; MMSE, Mini-Mental State Examination; MoCA, Montreal Cognitive Assessment; CDT, Clock Drawing Task; ADL, Activity of Daily Living Scale; HAMD, Hamilton Depression Scale; HIS, Hachinski Ischemic Score.

### Interhemispheric functional connectivity in the CN subjects

**Fig 1:**
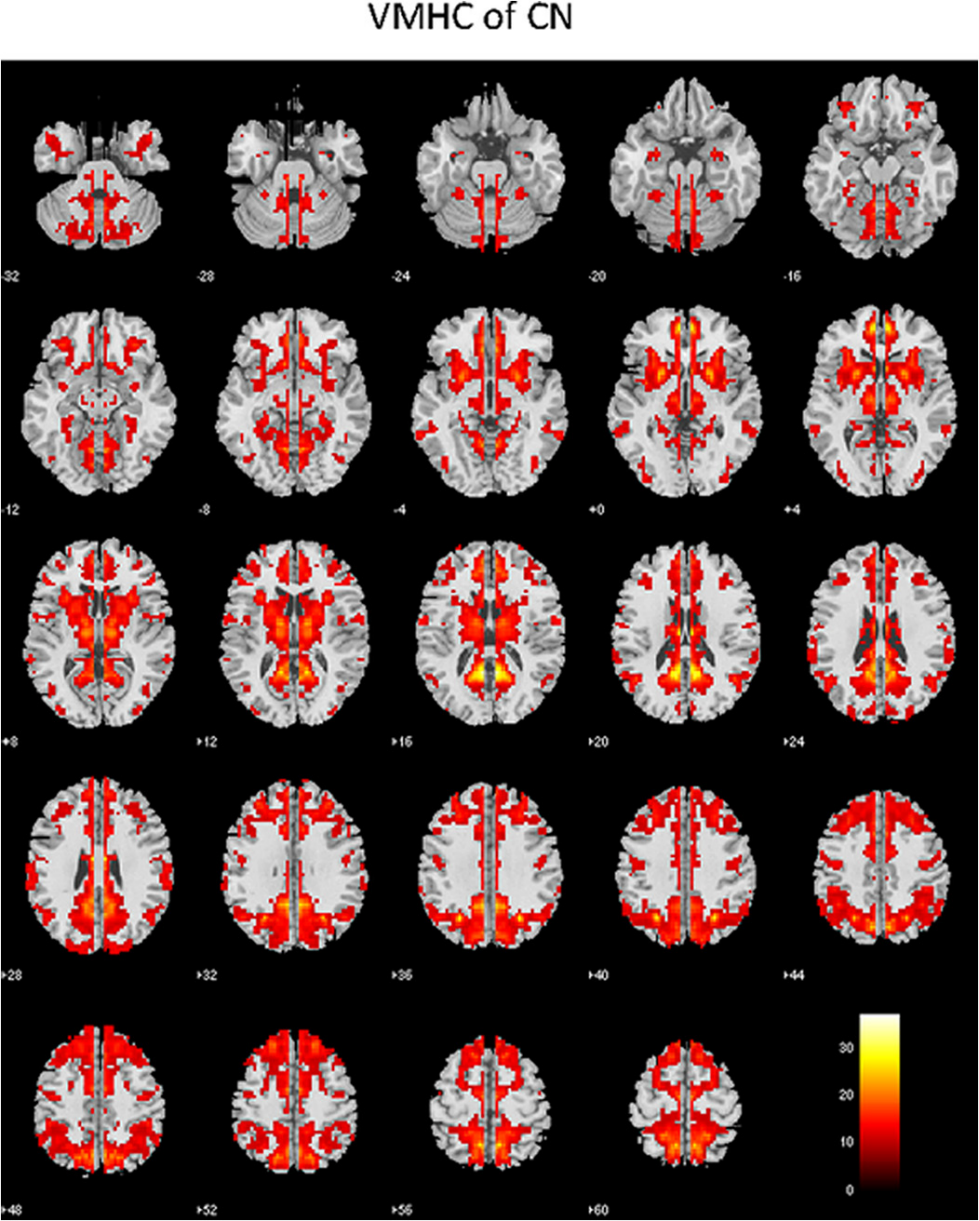
Normative VMHC map of the CN group (one-sample t-test, family-wise error corrected, p < 0.001, extent threshold = 10).

Fig 1 shows the VMHC map of the CN group representing the interhemispheric connectivity in the brain. Each pair of voxels symmetrically located on each hemisphere in the VMHC map represents the correlation coefficient between the time series of the rs-fMRI data from the two voxels. The VMHC map from CN revealed strong interhemispheric connectivities in the brain regions known to be important for cognition, including the anterior and posterior cingulate cortex (ACC and PCC), dorsolateral prefrontal cortex (DLPFC), orbitofrontal cortex (OFC), sensorimotor cortex (SMC), parietal and occipital cortices, hippocampus and various temporal cortices, insula, basal ganglia, and thalamus. This VMHC map from the CN cohort is consistent with those obtained by a previous study [25].

### Differences in interhemispheric functional connectivity among AD, MCI and CN

**Fig 2:**
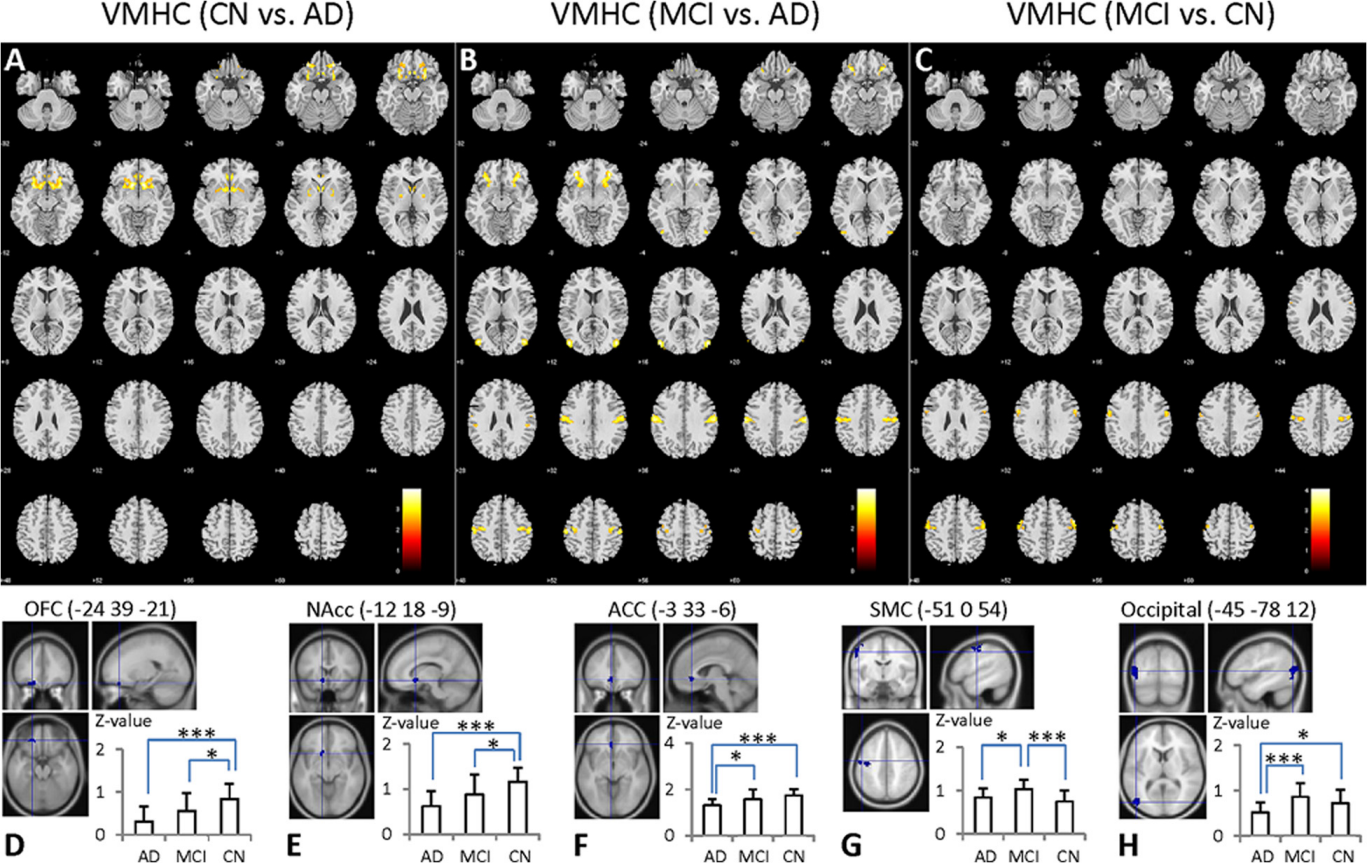
Cross-cohort comparisons of VMHC. **A**: The AD subjects showed significantly decreased VMHC in the OFC, ACC, POC, NAcc, putamen, caudate and insula compared to CN. **B**: The AD subjects showed significantly decreased VMHC in the OFC, putamen, caudate, insula, SMC, and OcG compared to MCI. **C**: The MCI subjects showed significantly increased VMHC in the SMC compared to CN. **D-H** show the locations of the five anatomical structures where the VMHC was significantly different across the three cohorts. The values in the bar graphs are z-scores transformed from the VMHC values. * represents statistical differences between groups (*, *p* ≤ 0.05; ***, *p* ≤ 0.001).

Fig 2 and Table 2 show the differences among the three cohorts by voxel-based comparisons of the VMHC maps. Compared to the CN subjects, the AD patients had significantly weaker VMHC in the OFC, ACC, nucleus accumbens (NAcc), primary olfactory cortex (POC), putamen, caudate, and insula, suggesting a reduction of interhemispheric connectivity in these structures in AD (Fig 2A). The AD patients also had significantly weaker VMHC than the MCI subjects in the OFC, putamen, caudate, insula, sensorimotor cortex (SMC), and occipital gyrus (OcG) (Fig 2B). In contrast, the MCI subjects had significantly stronger VMHC in the SMC than the CN subjects (Fig 2C).

**Table 2:** VMHC differences between AD, MCI and CN.

	Brain Regions	Cluster- size	Coordinates (MNI)			*t*-score	*p*-value corrected/ uncorrected
			x	y	z		
CN>AD	OFC/ ACC/ POC/ NAcc putamen/ caudate/ insula	591	-24	39	-21	4	0.000/0.000
MCI>CN	SMC (postcentral/ precentral gyrus)	116	-48	3	54	3.54	0.069/0.001
MCI>AD	OFC/ putamen/ caudate/ insula	109	-33	39	-9	3.75	0.091/0.001
	OcG (middle/ inferior occipital gyrus)	99	-45	-78	12	3.90	0.137/0.002
	SMC (postcentral/ precentral gyrus)	218	-60	-18	39	3.58	0.002/0.000

ANOVA, uncorrected, p < 0.01, extent threshold = 97. Coordinates are given in the left-posterior-inferior system. For the symmetric results, only the left side structures are listed. Abbreviations: MNI, Montreal Neurological Institute space; x, y, z, coordinates of cluster locations in the MNI space; OFC, orbitofrontal cortex; ACC, anterior cingulate cortex; NAcc, nucleus accumbens; POC, primary olfactory cortex; SMC, sensorimotor cortex; OcG, occipital gyrus.

To further demonstrate the above variations in AD and MCI, we extracted the VMHC from those regions that showed significant differences among the three cohorts (Fig 2D–2H). The OFC, ACC and NAcc showed a trend of gradual decreasing VMHC from CN to MCI and then to AD. However, in the regions of SMC and OcG, VMHC in MCIs appeared significantly stronger than those in both ADs and CNs.

### DTI and volume changes among AD, MCI and CN

**Fig 3:**
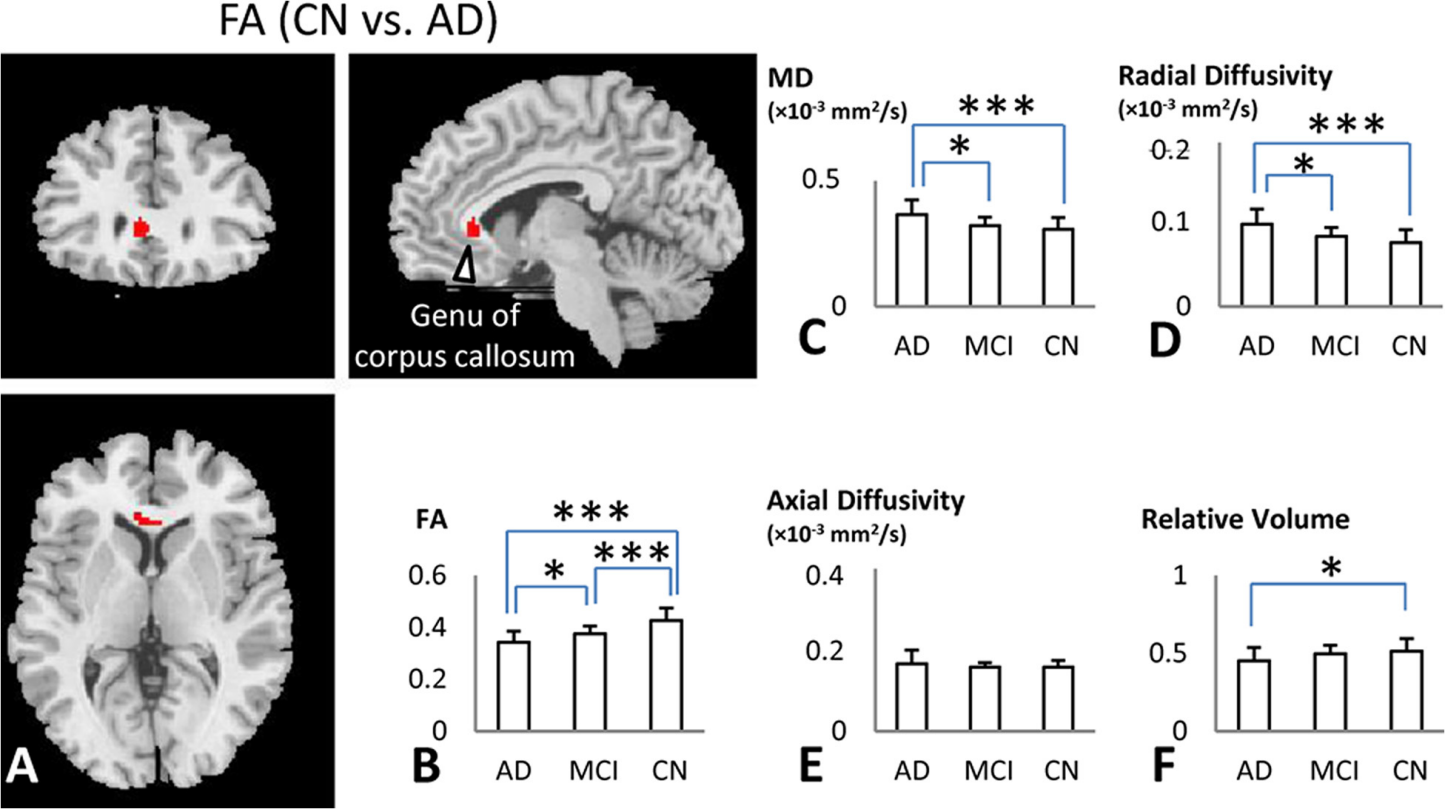
DTI and volumetric differences among AD, MCI and CN. **A:** Voxel-based analysis showed significant difference of FA between AD and CN in the genu of the corpus callosum (family-wise error corrected, *p* < 0.05, extent threshold = 10). **B-F:** Group comparisons revealed the patterns of diffusion parameters and volume changes in the genu of the corpus callosum (FA: AD < MCI < CN; MD: AD > CN / MCI; λ_┴_: AD > CN / MCI; λ_‖_: no difference; Volume: AD < CN/MCI). * represents statistical differences between groups (*, *p* ≤ 0.05; ***, *p* ≤ 0.001)

Voxel-based analysis detected significant difference in FA between CNs and ADs in the genu of the corpus callosum (Fig 3A). The reduction of FA in the genu of the corpus callosum presented a similar trend as seen in VMHC, i.e. AD < MCI < CN (Fig 3B). This trend of degeneration of the corpus callosum genu was supported by the significantly increased local MD and λ_┴_ (Fig 3C and 3D). There was no significant difference in λ_‖_ among any of the three groups (Fig 3E). We also found volume loss in the genu of the corpus callosum in the AD patients compared to CN (one-way ANOVA, *t* = -2.36, *p* = 0.02) (Fig 3F).

Table 3 shows significant positive correlations between VMHC in the anterior brain regions (OFC, ACC, and NAcc) and FA value of the genu of the corpus callosum. Additionally, the λ_┴_ value of the genu was negatively correlated with VMHC in the ACC.

**Table 3:** Correlation between diffusion parameters, volume of the genu of corpus callosum, VMHC, and MMSE scores of all the subjects.

	VMHC(OFC)/p-value	VMHC(ACC)/p-value	VMHC(NAcc)/p-value	MMSE /p-value
FA	0.404/0.005**	0.417/0.004**	0.45/0.002**	0.488/0.001***
MD	-0.155/0.299	-0.221/0.135	-0.108/0.470	-0.313/0.032*
λ‖	0.112/0.452	0.042/0.778	0.204/0.169	-0.110/0.943
λ_┴_	-0.288/0.124	-0.292/0.047*	-0.193/0.195	-0.382/0.008**
Volume	0.216/0.145	0.052/0.727	0.178/0.232	0.339/0.002*
VMHC(OFC)	-			0.447/0.002**
VMHC(ACC)	0.57/0.000***	-		0.442/0.002**
VMHC(NAcc)	0.613/0.000***	0.488/0.001***	-	0.418/0.003**
VMHC(SMC)	0.124/0.408	0.236/0.110	0.21/0.157	0.039/0.795
VMHC(OcG)	0.077/0.609	0.475/0.001***	0.245/0.097	0.388/0.007**

The values in the table are the correlation coefficients and corresponding p-values (partial correlation with age effect corrected).

*, p ≤ 0.05;

**, p ≤ 0.01;

***, p ≤ 0.001.

Abbreviations: FA, fractional anisotropy; MD, mean diffusivity (×10–3 mm2/s); λ‖, axial diffusivity (×10–3 mm2/s); λ┴, radial diffusion (×10–3 mm2/s); VMHC, voxel mirror homotopic connectivity; OFC, orbitofrontal cortex; ACC, anterior cingulate cortex; NAcc, nucleus accumbens; SMC, sensorimotor cortex; OcG, occipital gyrus.

### Relationship between interhemispheric connectivity and cognitive performance

There was no significant correlation between either VMHC or the diffusion parameters in the genu of corpus callosum and MMSE within each subject group (*r* ≤ 0.451, *p* ≥ 0.080). For all the subjects, the DTI parameters including FA, MD, λ_┴_ and volume in the genu of the corpus callosum were closely associated with MMSE scores (Table 3). The values of FA and volume in the genu of the corpus callosum were positively correlated with MMSE, while the values of MD and λ_┴_ were negatively correlated with MMSE scores. Consistent with the structural connectivity, the VMHCs in the brain regions of OFC, ACC, NAcc and OcG were positively correlated with MMSE scores.

## Discussion

### Major findings

By measuring the VMHC of rs-fMRI signals, we observed decreased VMHC in AD and MCI subjects in anterior brain regions including the prefrontal cortices and subcortical regions with a pattern of AD<MCI<CN. Increased VMHC was observed in the MCI in posterior brain regions with patterns of AD/CN < MCI (in SMC) and AD < CN/MCI (in OcG). In addition, DTI analysis among the three cohorts demonstrated that the most significant white matter change located in the genu of corpus callosum, which was revealed by the decreased FA and increased MD values in AD and MCI. Further analysis demonstrated that the VMHCs in the prefrontal cortices and subcortical regions were positively associated with the diffusion parameters in the genu of corpus callosum which demonstrated the association between structural and functional connectivity. Finally, the VMHCs in the above regions and diffusion parameters in the genu of corpus callosum showed significant correlations with the cognitive performance measured by the MMSE.

### Resting-state interhemispheric functional connectivity

The AD and MCI subjects showed decreased VMHC in the prefrontal brain regions (OFC, ACC and POC), subcortical regions (NAcc, putamen, and caudate nucleus) and insula compared with the CNs (Fig 2A and Table 2). The significant trend of gradually decreased VMHC, i.e. AD < MCI < CN, indicates a progressive deterioration in interhemispheric connection from MCI to AD.

The prefrontal brain regions are known to be involved in functional deficits at a very early stage of AD. For example, the OFC is involved in AD cognitive functional deficits (spatial construction, decision-making, and working memory) [35], and grey matter atrophy in the OFC has frequently been reported in AD patients [36–38]. The ACC plays a key role in cognitive control and emotion [39], which often present dysfunction in AD patients [40, 41]. The POC is the key structure for olfaction and also the early site of AD pathology. Olfactory deficits in odor detection, identification, and discrimination are prevalent in the early-stage of AD [42–44]. A recent study found that a combined olfactory test for odor detection, identification, and discrimination functions had a higher specificity than the hippocampal volume loss in the detection of AD [45]. Besides the above prefrontal regions, AD patients also presented decreased VMHC in the subcortical regions (NAcc, putamen, and caudate nucleus) and insula. These brain structures also play important roles in memory and learning processes [46–48]. Although the functions of these regions have been less investigated in AD, their structural abnormalities have been consistently reported in AD studies [49–52].

The hallmark AD pathology, neurofibrillary tangle and amyloid-β plaque deposition have been found in the OFC [53, 54], ACC [55], POC [56, 57], insula [58] and subcortical regions [59] at early stages of the disease. This suggests that these regions are preferentially vulnerable to the early attack from the disease, which is consistent with AD pathologic progression patterns [60]. Our results extended the findings in previous studies and provided new evidence that VMHC could be a sensitive measure for the interhemispheric functional connectivity for AD studies.

The MCI subjects had significantly stronger VMHC in the SMC than the CN subjects (Fig 2B and 2C and Table 2). The VMHC increase in the SMC of the MCI group may reflect a possible recruitment in hemispheric cooperation as a compensatory mechanism [20,60,61]. It has been shown that older adults commonly recruit more sensorimotor regions to counteract the neurobiological changes due to aging [62]. One previous fMRI study using the VMHC method has demonstrated an increased interhemispheric connectivity in sensorimotor regions during aging [20]. Previous studies have shown that bimanual motor coordination in older adults is associated with increased functional brain connectivity [61–63]. One magnetoencephalography study also showed higher interhemispheric synchronization in MCI subjects than in healthy controls [64]. Our results are in agreement with the literature. Taking together such studies with different modalities, we hypothesize that MCI subjects, challenged by the pathological degenerations in the prefrontal brain areas, would strengthen interhemispheric cooperation of SMC regions to resist the cognitive decline. It may be a similar mechanism happens in the OcG. Before developing to AD, the MCI subjects might be able to recruit additional neural resources in the posterior region of OcG that are unaffected or less-affected by the disease, to compensate for interhemispheric disconnections in the disease-affected frontal brain regions. In addition, according to the Table 3, the interhemispheric RSFCs of the OcG were positively correlated with the cognitive performance as measured by MMSE, which suggested that stronger inter-hemispheric connectivity of the OcG contributed to the better cognitive performance, supporting the compensatory mechanism. In the study, we didn’t find the significant correlation between the SMC regions and the MMSE, we speculated that the sample size may be too small to find a robust association, In the future, to further study the underlying mechanism for this increased VMHC in the MCI, we will perform a longitudinal study of large sample of MCI subjects to provide the more valuable information to further confirm the compensatory mechanism in these regions.

It is known that the earliest regions of AD pathology are located in the medial temporal and parietal cortexes, which are involved in the default mode network. It is worthy to mention that the analytic approaches (i.e., VMHC) used in the current study are very different from those previously applied. Previous fMRI studies in AD consistently discussed brain functional connectivity (i.e., default mode network) based on whole brain connectivity, i.e., all voxels are correlated with each other. However, in the current study, we focused on the interhemispheric functional connectivity by measuring the voxel-mirrored homotopic connectivity. For the first time, we applied this method to AD study and provided the spatial pattern of the intrinsic interhemispheric connectivity changes across AD, MCI, and CN.

### Corpus callosum degeneration and its correlation with VMHC changes

The pathological mechanisms underlying the VMHC deficits could be related to widespread white matter integrity abnormalities observed in AD patients. We found the most significant white matter degeneration was located in the genu of the corpus callosum (Fig 3). Significant corpus callosum atrophy has been reported in AD and MCI [3, 4, 65]. Myelin breakdown is regarded as the primary process of leading to white matter damage in AD [66]. Recent data indicate myelin can be directly damaged by oligomerized amyloid-β plaques that are an important early event in the pathogenesis of AD. Furthermore, homeostatic responses to this myelin breakdown increase intracortical toxicity, which might explain the progressive neuronal damage in AD [26, 66, 67]. The distribution of myelin breakdown is not global. Rather, the regions that are most susceptible to pathological changes in AD are the late myelinated regions, which have fewer oligodendrocytes supporting a greater numbers of axons [26, 68, 69]. The myelin breakdown process in AD affects the late-myelinated anterior callosal subregions, resulting in a significant atrophy of the genu of the corpus callosum in AD patients [5, 70]. Our findings of FA, MD and λ_┴_ abnormalities in the genu of the corpus callosum in AD support the myelin breakdown retrogenesis hypothesis.

It is interesting that the VMHCs in the prefrontal (OFC, ACC) and subcortical regions (NAcc) were positively correlated with the FA values of the genu of the corpus callosum (Table 3). Since the prefrontal regions between the two hemispheres communicate through the genu of the corpus callosum [71], it was not an accident that white matter integrity was impaired in this region of AD patients, which could disrupt the functional connectivities between prefrontal homotopic regions. Previous studies had provided significant evidence of the robust links between structural and functional connectivity in the human brain [72–74]. A previous study demonstrated a prominent loss of interhemispheric BOLD correlations after complete sectioning of the corpus callosum [75]. Another study confirmed that lack of normal callosal development can lead to deficits in functional connectivity related to cognitive impairments [76]. It is plausible to use the white matter integrity abnormalities in the genu of the corpus callosum to explain the reduction of VMHC in the prefrontal regions of AD and MCI. This direct correlation obtained by the two entirely different imaging modalities provided a mutual support of the findings in the functional and structural alterations in AD.

### Relationship between interhemispheric connectivity and cognitive performance

The correlation analysis of all the subjects demonstrated that the interhemispheric RSFCs in specific brain regions and DTI parameters in the genu of corpus callosum were positively correlated with the cognitive performance as measured by the MMSE. Since we did not observe significant correlation in each subject group, the observed correlation of all the subjects may be dominated by the group differences. Thus the correlation of reduced interhemispheric functional and structural connectivity with the cognitive decline in AD needs to be further studied. The brain relies on interhemispheric information transfer for mediating cognition function, which is subserved by the corpus callosum [77]. Previous studies have shown that even subtle degradation of the corpus callosum in neurologically impaired patients can be related to deficits in the transfer of information between the hemispheres [78]. Based on the above statement, the bilateral prefrontal regions (OFC and ACC), which are connected by the genu of corpus callosum, played a pivotal role in integrating information and mediating cognitive functions.

### Further consideration

There are several technical issues need to be addressed. First, we could not exclude the interference of some potential confounding factors, such as respiratory and cardiac cycle artifacts, and slow sampling rates. It is known that slow sampling rates (as in this study TR = 2 s), noise from the cardiac and respiratory cycle can alias into the resting-state low frequency ranges. Second, the DTI acquisition and post-processing methods can be improved in the future. For example, the spatial resolution, partial-volume effects, noises/artifacts, and uncorrected distortions may influence the result. In addition, this is a first exploration to see if there is any association between reduced white matter integrity & VMHC. Our findings revealed the close relationship between diffusion changes in the genu of corpus callosum and VMHC changes in the MCI and AD. It would be better to analyze the anatomical connectivity between homotopic voxels to clarify the direct link between VMHC and DTI abnormalities. Future research will further investigate the relationship between these modalities. Finally, the sample size is small and the findings may be altered in large populations. Thus a longitudinal study of large sample of MCI subjects will be beneficial to elucidate the impact of the disease on interhemispheric connectivity, which may in turn provide a valuable imaging marker for the diagnosis of AD.

## Conclusions

In this study we demonstrated significant changes of interhemispheric functional connectivity in the AD and MCI using VMHC analysis. The interhemispheric functional connectivity decline in the OFC, ACC, and NAcc was significantly correlated with the structural degeneration in the genu of the corpus callosum. These results suggest that VMHC can be used as a biomarker for the degeneration of the interhemispheric connectivity in AD.

## References

[1] Petersen RC, Doody R, Kurz A, Mohs RC, Morris JC, Rabins PV, et al Current concepts in mild cognitive impairment. Archives of neurology. 2001;58:1985–1992. 11735772 10.1001/archneur.58.12.1985

[2] Lakmache Y, Lassonde M, Gauthier S, Frigon JY, Lepore F. Interhemispheric disconnection syndrome in Alzheimer's disease. Proc Natl Acad Sci U SA.1998;95: 9042–9046. 9671801 10.1073/pnas.95.15.9042 PMC21199

[3] Chaim TM, Duran FL, Uchida RR, Périco CA, de Castro CC, Busatto GF, et al Volumetric reduction of the corpus callosum in Alzheimer's disease in vivo as assessed with voxel-based morphometry. Psychiatry Res. 2007;154:59–68. 17174533 10.1016/j.pscychresns.2006.04.003

[4] Wang PJ, Saykin AJ, Flashman LA, Wishart HA, Rabin LA, Santulli RB, et al Regionally specific atrophy of the corpus callosum in AD, MCI and cognitive complaints. Neurobiol Aging. 2006;27:1613–1617. 16271806 10.1016/j.neurobiolaging.2005.09.035 PMC3482483

[5] Di Paola M, Di Iulio F, Cherubini A, Blundo C, Casini AR, Sancesario G, et al When, where, and how the corpus callosum changes in MCI and AD: a multimodal MRI study. Neurology. 2010;74:1136–1142. 10.1212/WNL.0b013e3181d7d8cb 20368633

[6] Preti MG, Baglio F, Laganà MM, Griffanti L, Nemni R, Clerici M, et al Assessing corpus callosum changes in Alzheimer's disease: comparison between tract-based spatial statistics and atlas-based tractography. PLoS One. 2012;7: e35856 10.1371/journal.pone.0035856 22545143 PMC3335803

[7] Xie S, Xiao JX, Gong GL, Zang YF, Wang YH, Wu HK, et al Voxel-based detection of white matter abnormalities in mild Alzheimer disease. Neurology. 2006;66:1845–1849. 16801648 10.1212/01.wnl.0000219625.77625.aa

[8] Greicius MD, Supekar K, Menon V, Dougherty RF. Resting-state functional connectivity reflects structural connectivity in the default mode network. Cereb Cortex. 2009;19:72–78. 10.1093/cercor/bhn059 18403396 PMC2605172

[9] Wang L, Zang Y, He Y, Liang M, Zhang X, Tian L, et al Changes in hippocampal connectivity in the early stages of Alzheimer's disease: evidence from resting state fMRI. Neuroimage. 2006;31:496–504. 16473024 10.1016/j.neuroimage.2005.12.033

[10] Allen G, Barnard H, McColl R, Hester AL, Fields JA, Weiner MF, et al Reduced hippocampal functional connectivity in Alzheimer disease. Arch Neurol. 2007;64:1482–1487. 17923631 10.1001/archneur.64.10.1482

[11] Bai F, Watson DR, Yu H, Shi Y, Yuan Y, Zhang Z, et al Abnormal resting-state functional connectivity of posterior cingulate cortex in amnestic type mild cognitive impairment. Brain Res. 2009;1302:167–174. 10.1016/j.brainres.2009.09.028 19765560

[12] Zhang HY, Wang SJ, Liu B, Ma ZL, Yang M, Zhang ZJ, et al Resting brain connectivity: changes during the progress of Alzheimer disease. Radiology. 2010;256:598–606. 10.1148/radiol.10091701 20656843

[13] Zhang HY, Wang SJ, Xing J, Liu B, Ma ZL, Yang M, et al Detection of PCC functional connectivity characteristics in resting-state fMRI in mild Alzheimer's disease. Behav Brain Res. 2009;197:103–108. 10.1016/j.bbr.2008.08.012 18786570

[14] Greicius MD, Srivastava G, Reiss AL, Menon V. Default-mode network activity distinguishes Alzheimer's disease from healthy aging: evidence from functional MRI. Proc Natl Acad Sci U S A. 2004;101: 4637–4642. 15070770 10.1073/pnas.0308627101 PMC384799

[15] Sorg C, Riedl V, Mühlau M, Calhoun VD, Eichele T, Laer L, et al Selective changes of resting-state networks in individuals at risk for Alzheimer's disease. Proc Natl Acad Sci U S A. 2007;104:18760–18765. 18003904 10.1073/pnas.0708803104 PMC2141850

[16] Biswal B, Yetkin FZ, Haughton VM, Hyde JS. Functional connectivity in the motor cortex of resting human brain using echo-planar MRI. Magn Reson Med.1995;34: 537–541. 8524021 10.1002/mrm.1910340409

[17] Fox MD, Raichle ME. Spontaneous fluctuations in brain activity observed with functional magnetic resonance imaging. Nat Rev Neurosci. 2007;8:700–711. 17704812 10.1038/nrn2201

[18] Salvador R, Martínez A, Pomarol-Clotet E, Gomar J, Vila F, Sarró S, et al A simple view of the brain through a frequency-specific functional connectivity measure. Neuroimage.2008;39:279–289. 17919927 10.1016/j.neuroimage.2007.08.018

[19] Stark DE, Margulies DS, Shehzad ZE, Reiss P, Kelly AM, Uddin LQ, et al Regional variation in interhemispheric coordination of intrinsic hemodynamic fluctuations. J Neurosci. 2008;28:13754–13764. 10.1523/JNEUROSCI.4544-08.2008 19091966 PMC4113425

[20] Zuo XN, Kelly C, Di Martino A, Mennes M, Margulies DS, Bangaru S, et al Growing together and growing apart: regional and sex differences in the lifespan developmental trajectories of functional homotopy. J Neurosci. 2010;30:15034–15043. 10.1523/JNEUROSCI.2612-10.2010 21068309 PMC2997358

[21] Anderson JS, Druzgal TJ, Froehlich A, DuBray MB, Lange N, Alexander AL, et al Decreased interhemispheric functional connectivity in autism. Cereb Cortex. 2011;21:1134–1146. 10.1093/cercor/bhq190 20943668 PMC3077433

[22] Kelly C, Zuo XN, Gotimer K, Cox CL, Lynch L, Brock D, et al Reduced interhemispheric resting state functional connectivity in cocaine addiction. Biol Psychiatry. 2011;69:684–692. 10.1016/j.biopsych.2010.11.022 21251646 PMC3056937

[23] Hoptman MJ, Zuo XN, D'Angelo D, Mauro CJ, Butler PD, Milham MP, et al Decreased interhemispheric coordination in schizophrenia: a resting state fMRI study. Schizophr Res. 2012;141:1–7. 10.1016/j.schres.2012.07.027 22910401 PMC3446206

[24] Zhou Y, Milham M, Zuo XN, Kelly C, Jaggi H, Herbert J, et al Functional Homotopic Changes in Multiple Sclerosis with Resting-State Functional MR Imaging. AJNR Am J Neuroradiol. 2013;34:1180–1187. 10.3174/ajnr.A3386 23348760 PMC3707620

[25] Wang L, Li K, Zhang QE, Zeng YW, Jin Z, Dai WJ. Interhemispheric functional connectivity and its relationships with clinical characteristics in major depressive disorder: a resting state fMRI study. PLoS One. 2013;8:e60191 10.1371/journal.pone.0060191 23555920 PMC3612036

[26] Bartzokis G, Cummings JL, Sultzer D, Henderson VW, Nuechterlein KH, Mintz J, et al White matter structural integrity in healthy aging adults and patients with Alzheimer disease: a magnetic resonance imaging study. Arch Neurol. 2003;60: 393–398. 12633151 10.1001/archneur.60.3.393

[27] American Psychiatric Association. Diagnostic and statistical manual of mental disorders (DSM-IV) Washington, DC: American Psychiatric Association Press;1994.

[28] McKhann G, Drachman D, Folstein M, Katzman R, Price D, Stadlan EM, et al Clinical diagnosis of Alzheimer's disease: report of the NINCDS-ADRDA Work Group under the auspices of Department of Health and Human Services Task Force on Alzheimer's Disease. Neurology.1984;34:939–944. 6610841 10.1212/wnl.34.7.939

[29] Morris JC. The Clinical Dementia Rating (CDR): current version and scoring rules. Neurology. 1993;43:2412–2414. 8232972 10.1212/wnl.43.11.2412-a

[30] Petersen RC, Smith GE, Waring SC, Ivnik RJ, Tangalos EG, Kokmen E, et al Mild cognitive impairment: clinical characterization and outcome. Arch Neurol.1999;56: 303–308. 10190820 10.1001/archneur.56.3.303

[31] Petersen RC, Stevens JC, Ganguli M, Tangalos EG, Cummings JL, DeKosky ST. Practice parameter: Early detection of dementia: Mild cognitive impairment (an evidence-based review) Report of the Quality Standards Subcommittee of the American Academy of Neurology. Neurology. 2001;56:1133–1142. 11342677 10.1212/wnl.56.9.1133

[32] Albert MS, DeKosky ST, Dickson D, Dubois B, Feldman HH, Fox NC, et al The diagnosis of mild cognitive impairment due to Alzheimer's disease: Recommendations from the National Institute on Aging-Alzheimer‘s Association workgroups on diagnostic guidelines for Alzheimer’s disease. Alzheimer's & Dementia. 2011;7:270–279. 10.1016/j.jalz.2011.03.008 PMC3312027 21514249

[33] Yan C, Zang Y. DPARSF: A MATLAB Toolbox for "Pipeline" Data Analysis of Resting-State fMRI. Front Syst Neurosci. 2010;4:13 10.3389/fnsys.2010.00013 20577591 PMC2889691

[34] Stadlbauer A, Salomonowitz E, Strunk G, Hammen T, Ganslandt O. Age-related degradation in the central nervous system: assessment with diffusion-tensor imaging and quantitative fiber tracking. Radiology.2008;247:179–188. 10.1148/radiol.2471070707 18292477

[35] Van Hoesen GW, Parvizi J, Chu CC. Orbito frontal cortex pathology in Alzheimer's disease. Cereb Cortex. 2000;10:243–251. 10731219 10.1093/cercor/10.3.243

[36] Plant C, Teipel SJ, Oswald A, Böhm C, Meindl T, Mourao-Miranda J, et al Automated detection of brain atrophy patterns based on MRI for the prediction of Alzheimer's disease. Neuroimage. 2010;50:162–174. 10.1016/j.neuroimage.2009.11.046 19961938 PMC2838472

[37] Wang Z, Yan C, Zhao C, Qi Z, Zhou W, Lu J, et al Spatial patterns of intrinsic brain activity in mild cognitive impairment and alzheimer's disease: A resting-state functional MRI study. Hum Brain Mapp. 2011;32:1720–1740. 10.1002/hbm.21140 21077137 PMC6870362

[38] Tondelli M, Wilcock GK, Nichelli P, De Jager CA, Jenkinson M, Zamboni G, et al Structural MRI changes detectable up to ten years before clinical Alzheimer's disease. Neurobiol Aging. 2012;33:25–36. 10.1016/j.neurobiolaging.2011.05.018 21782287

[39] Bush G, Luu P, Posner MI. Cognitive and emotional influences in anterior cingulate cortex. Trends Cogn Sci. 2000;4:215–222. 10827444 10.1016/s1364-6613(00)01483-2

[40] Agosta F, Pievani M, Geroldi C, Copetti M, Frisoni GB, Filippi M, et al Resting state fMRI in Alzheimer's disease: beyond the default mode network. Neurobiol Aging. 2012;33:1564–1578. 10.1016/j.neurobiolaging.2011.06.007 21813210

[41] Brier MR, Thomas JB, Snyder AZ, Benzinger TL, Zhang D, Raichle ME, et al Loss of intranetwork and internetwork resting state functional connections with Alzheimer's disease progression. J Neurosci. 2012;32:8890–8899. 10.1523/JNEUROSCI.5698-11.2012 22745490 PMC3458508

[42] Doty RL, Reyes PF, Gregor T. Presence of both odor identification and detection deficits in Alzheimer's disease. Brain Res Bull.1987;18:597–600. 3607528 10.1016/0361-9230(87)90129-8

[43] Mesholam RI, Moberg PJ, Mahr RN, Doty RL. Olfaction in neurodegenerative disease: a meta-analysis of olfactory functioning in Alzheimer's and Parkinson's diseases. Arch Neurol. 1998;55:84–90. 9443714 10.1001/archneur.55.1.84

[44] Murphy C. Loss of olfactory function in dementing disease. Physiol Behav.1999; 66:177–182. 10336141 10.1016/s0031-9384(98)00262-5

[45] Marigliano V, Gualdi G, Servello A, Marigliano B, Volpe LD, Fioretti A, et al Olfactory Deficit and Hippocampal Volume Loss for Early Diagnosis of Alzheimer Disease: A Pilot Study. Alzheimer Dis Assoc Disord. 2014;28:194–197. 10.1097/WAD.0b013e31827bdb9f 23314063

[46] Xie C, Bai F, Yu H, Shi Y, Yuan Y, Chen G, et al Abnormal insula functional network is associated with episodic memory decline in amnestic mild cognitive impairment. Neuroimage. 2012;63:320–327. 10.1016/j.neuroimage.2012.06.062 22776459 PMC4513936

[47] González-Burgos I, Feria-Velasco A. Serotonin/dopamine interaction in memory formation. Prog Brain Res.2008;172: 603–623. 10.1016/S0079-6123(08)00928-X 18772052

[48] Graybiel AM. Habits, rituals, and the evaluative brain. Annu Rev Neurosci. 2008;31:359–387. 10.1146/annurev.neuro.29.051605.112851 18558860

[49] Guo X, Han Y, Chen K, Wang Y, Yao L. Mapping joint grey and white matter reductions in Alzheimer's disease using joint independent component analysis. Neurosci Lett. 2012;531:136–141. 10.1016/j.neulet.2012.10.038 23123779 PMC3652975

[50] de Jong LW, Ferrarini L, van der Grond J, Milles JR, Reiber JH, Westendorp RG, et al Shape abnormalities of the striatum in Alzheimer's disease. J Alzheimers Dis. 2011;23:49–59. 10.3233/JAD-2010-101026 20930298

[51] Hall AM, Moore RY, Lopez OL, Kuller L, Becker JT. Basal forebrain atrophy is a presymptomatic marker for Alzheimer's disease. Alzheimers Dement. 2008;4: 271–279. 10.1016/j.jalz.2008.04.005 18631978 PMC2517158

[52] Teipel SJ, Flatz WH, Heinsen H, Bokde AL, Schoenberg SO, Stockel S, et al Measurement of basal forebrain atrophy in Alzheimer's disease using MRI. Brain. 2005;128:2626–2644 16014654 10.1093/brain/awh589

[53] Resnick SM, Lamar M, Driscoll I. Vulnerability of the orbitofrontal cortex to age-associated structural and functional brain changes. Ann N Y Acad Sci. 2007; 1121: 562–575. 17846159 10.1196/annals.1401.027

[54] Koivunen J, Verkkoniemi A, Aalto S, Paetau A, Ahonen JP, Viitanen M, et al PET amyloid ligand [11C] PIB uptake shows predominantly striatal increase in variant Alzheimer's disease. Brain. 2008;131:1845–1853. 10.1093/brain/awn107 18583368

[55] Van Hoesen GW, Parvizi J, Chu CC. Orbitofrontal cortex pathology in Alzheimer's disease. Cereb Cortex. 2000;10:243–251. 10731219 10.1093/cercor/10.3.243

[56] Attems J, Jellinger KA. Olfactory tau pathology in Alzheimer disease and mild cognitive impairment. Clin Neuropathol. 2006;25:265–271. 17140156

[57] Wesson DW, Levy E, Nixon RA, Wilson DA. Olfactory dysfunction correlates with amyloid-beta burden in an Alzheimer's disease mouse model. J Neurosci. 2010; 30: 505–514. 10.1523/JNEUROSCI.4622-09.2010 20071513 PMC2826174

[58] Villemagne VL, Burnham S, Bourgeat P, Brown B, Ellis KA, Salvado O, et al Amyloid beta deposition, neurodegeneration, and cognitive decline in sporadic Alzheimer's disease: a prospective cohort study. Lancet Neurol. 2013;12: 357–367. 10.1016/S1474-4422(13)70044-9 23477989

[59] Selden N, Mesulam MM, Geula C. Human striatum: the distribution of neurofibrillary tangles in Alzheimer's disease. Brain Res.1994;648:327–331. 7922549 10.1016/0006-8993(94)91136-3

[60] Braak H, Braak E. Neuropathological stageing of Alzheimer-related changes. Acta Neuropathol.1991;82:239–259. 1759558 10.1007/BF00308809

[61] Donchin O, Gribova A, Steinberg O, Bergman H, Vaadia E. Primary motor cortex is involved in bimanual coordination. Nature.1998;395:274–278. 9751054 10.1038/26220

[62] Marion SD, Kilian SC, Naramor TL, Brown WS. Normal development of bimanual coordination: visuomotor and interhemispheric contributions. Dev Neuropsychol. 2003;23:399–421. 12740193 10.1207/S15326942DN2303_6

[63] Heitger MH, Goble DJ, Dhollander T, Dupont P, Caeyenberghs K, Leemans A, et al Bimanual motor coordination in older adults is associated with increased functional brain connectivity—a graph-theoretical analysis. PLoS One. 2013;8: e62133 10.1371/journal.pone.0062133 23637982 PMC3639273

[64] Bajo R, Maestú F, Nevado A, Sancho M, érrez R, Campo P, et al Functional connectivity in mild cognitive impairment during a memory task: implications for the disconnection hypothesis. J Alzheimers Dis. 2010; 22:183–193. 10.3233/JAD-2010-100177 20847450

[65] Wang Z, Guo X, Qi Z, Yao L, Li K. Whole-brain voxel-based morphometry of white matter in mild cognitive impairment. Eur J Radiol. 2010;75:129–133. 10.1016/j.ejrad.2009.04.041 19443157

[66] Bartzokis G, Sultzer D, Lu PH, Nuechterlein KH, Mintz J, Cummings JL, et al Heterogeneous age-related breakdown of white matter structural integrity: implications for cortical "disconnection" in aging and Alzheimer's disease. Neurobiol Aging. 2004;25:843–851. 15212838 10.1016/j.neurobiolaging.2003.09.005

[67] Bartzokis G, Lu PH, Geschwind DH, Tingus K, Huang D, Mendez MF, et al Apolipoprotein E affects both myelin breakdown and cognition: implications for age-related trajectories of decline into dementia. Biol Psychiatry. 2007;62:1380–1387. 17659264 10.1016/j.biopsych.2007.03.024

[68] Braak H, Braak E. Development of Alzheimer-related neurofibrillary changes in the neocortex inversely recapitulates cortical myelogenesis. Acta Neuropathol. 1996;92:197–201. 8841666 10.1007/s004010050508

[69] Braak H, Del Tredici K, Schultz C, Braak E. Vulnerability of select neuronal types to Alzheimer's disease. Ann N Y Acad Sci. 2000;924:53–61. 11193802 10.1111/j.1749-6632.2000.tb05560.x

[70] Di Paola M, Spalletta G, Caltagirone C. In vivo structural neuroanatomy of corpus callosum in Alzheimer's disease and mild cognitive impairment using different MRI techniques: a review. J Alzheimers Dis. 2010;20:67–95. 10.3233/JAD-2010-1370 20164572

[71] van der Knaap LJ, van der Ham IJ. How does the corpus callosum mediate interhemispheric transfer? A review. Behav Brain Res. 2011;223:211–221. 10.1016/j.bbr.2011.04.018 21530590

[72] Hermundstad AM, Bassett DS, Brown KS, Aminoff EM, Clewett D, Freeman S, et al Structural foundations of resting-state and task-based functional connectivity in the human brain. Proc Natl Acad Sci U S A. 2013;110:6169–6174. 10.1073/pnas.1219562110 23530246 PMC3625268

[73] van den Heuvel MP, Mandl RC, Kahn RS, Hulshoff Pol HE. Functionally linked resting-state networks reflect the underlying structural connectivity architecture of the human brain. Hum Brain Mapp. 2009;30:3127–3141. 10.1002/hbm.20737 19235882 PMC6870902

[74] Greicius MD, Supekar K, Menon V, Dougherty RF. Resting-state functional connectivity reflects structural connectivity in the default mode network. Cereb Cortex. 2009;19:72–78. 10.1093/cercor/bhn059 18403396 PMC2605172

[75] Johnston JM, Vaishnavi SN, Smyth MD, Zhang D, He BJ, Zempel JM, et al Loss of resting interhemispheric functional connectivity after complete section of the corpus callosum. J Neurosci. 2008;28:6453–6458. 10.1523/JNEUROSCI.0573-08.2008 18562616 PMC2738991

[76] Hinkley LB, Marco EJ, Findlay AM, Honma S, Jeremy RJ, Strominger Z, et al The role of corpus callosum development in functional connectivity and cognitive processing. PLoS One. 2012;7:e39804 10.1371/journal.pone.0039804 22870191 PMC3411722

[77] Doron KW, Gazzaniga MS. Neuroimaging techniques offer new perspectives on callosal transfer and interhemispheric communication. Cortex. 2008; 44: 1023–1129. 10.1016/j.cortex.2008.03.007 18672233

[78] Schulte T, Muller-Oehring EM. Contribution of callosal connections to the interhemispheric integration of visuomotor and cognitive processes. Neuropsychol Rev. 2010;20:174–190. 10.1007/s11065-010-9130-1 20411431 PMC3442602

